# A scoping review of over-the-counter products for depression, anxiety and insomnia in older people

**DOI:** 10.1186/s12906-024-04585-0

**Published:** 2024-07-20

**Authors:** Rachael Frost, Silvy Mathew, Verity Thomas, Sayem Uddin, Adriana Salame, Christine Vial, Tanya Cohen, Sukvinder Kaur Bhamra, Juan Carlos Bazo Alvarez, Cini Bhanu, Michael Heinrich, Kate Walters

**Affiliations:** 1 Department of Primary Care and Population Health, University College London, London, UK; 2 UCL Medical School, University College London, London, UK; 3 Division of Medicine, University College London, London, UK; 4 Public Contributor, London, UK; 5 Medway School of Pharmacy, University of Kent, Kent, UK; 6 School of Pharmacy, University College London, London, UK; 7 China Medical University, Taichung, Taiwan; 8 School of Public and Allied Health, Faculty of Health, Liverpool John Moores University, 312 Tithebarn Building, Tithebarn Street, Liverpool, L2 2ER UK

**Keywords:** Scoping review, Medicine, Herbal medicine, Dietary supplements, Insomnia, Depression

## Abstract

**Background:**

Depression, anxiety, and insomnia are prevalent in older people and are associated with increased risk of mortality, dependency, falls and reduced quality of life. Prior to or whilst seeking treatment, older people often manage these symptoms or conditions using products purchased over the counter (OTC), such as medication or herbal products. This review aims to map the evidence available for OTC medications, herbal medicines and dietary supplements for depression, anxiety and insomnia in older adults.

**Methodology:**

We carried out a scoping review, including searches of five databases to identify relevant randomised controlled trials (inception-Dec 2022). We took an inclusive approach to products to represent the wide range that may be available online. Trials were summarised according to condition and product.

**Results:**

We included 47 trials and 10 ongoing trial protocols. Most targeted insomnia (*n* = 25), followed by depression (*n* = 20), and mixed conditions (*n* = 2). None evaluated products targeted at anxiety alone. Where reported, most products appeared to be safe for use, but studies rarely included people with multiple comorbidities or taking concomitant medication. Some types of melatonin for insomnia (*n* = 19) and omega-3 fatty acids for depression (*n* = 7) had more substantive evidence compared to the other products.

**Conclusion:**

There is a substantial gap in evidence for OTC products for anxiety in older people. This should be addressed in future trials. Research should also focus on products that are widely used, and these need to be tested in older populations that are similar to those who would use them in practice.

**Supplementary Information:**

The online version contains supplementary material available at 10.1186/s12906-024-04585-0.

## Key points

We carried out a scoping review of over-the-counter products for depression, anxiety and insomnia in older people. Out of 47 trials and 10 ongoing protocols, most studies targeted depression or insomnia - none targeted anxiety alone. Few products had more than one trial available, apart from melatonin and omega-3s, and studies rarely included people with multiple comorbidities or taking concomitant medication.

**Supplementary Information:**

The online version contains supplementary material available at 10.1186/s12906-024-04585-0.

## Introduction

 Depression, anxiety and insomnia are common in later life, which often involves challenges such as bereavement, chronic diseases, lack of family support and financial instability [1]. Estimates suggest that 7% of older people have major depressive disorder [2], 10% have insomnia [3] and 14% have an anxiety disorder [4]; and that the prevalence of these is increasing in the UK [5, 6]. These conditions are in turn associated with adverse outcomes, such as reduced quality of life [6] and increased risk of physical frailty [7, 8], dementia [9, 10] and mortality [11], and increased use of health services [12].

Current treatments are not optimal, as antidepressants such as selective serotonin reuptake inhibitors (SSRIs) have limited acceptability in older people [13], with negligible effects and an increased falls risk [14]. Similarly, Z-drugs for insomnia (such as zopiclone) are associated with an increased falls and fracture risk when used long term [15]. Although psychological therapies are effective for older people [16, 17], uptake rates are only half of those expected in the UK [18], with multiple access barriers, including local availability and perceptions that older people do not want them [19]. Further barriers exist for internet-based psychological approaches, such as lack of awareness of what is available, limited technological skills and preferences for face-to-face contact for mental health problems in order to build trust [20, 21]. For mild-moderate depressive symptoms and insomnia, evidence suggests older people prefer to self-manage [13, 22], with less surety on managing anxiety [23]. This may be prior to or whilst seeking GP support [24].

Using over the counter products (OTCs) with a claim for treating or preventing depression, anxiety, and insomnia, such as St John’s Wort (*Hypericum perforatum* L.) or antihistamines, is a key part of this self-management. UK sales of OTC products were £3.2 billion pounds in 2022, with £461 million spent on vitamins and minerals and £63 million on sleep-related products [25, 26]. In a US cohort study of older people, 38% used OTC medication and 64% used dietary supplements for any reason, with 47% of those taking multiple supplements simultaneously [27]. Specifically in insomnia, another survey found 21.9% older people used an OTC medication to improve sleep and 12.5% used herbal medicines, in comparison to 8.3% using prescription medication [28], but did not specify the products. Other surveys of older people focus on products taken rather than for which condition(s) products are taken. From these, commonly used products with a potential indication for mental health include valerian, Nytol herbal (hops, valerian, and passionflower), cod liver oil, multivitamins, vitamin D, gingko (*Ginkgo biloba* L.), turmeric (*Curcuma longa* L.), black seed oil (*Nigella sativa* L.) and cinnamon (*Cinnamomum* spp.) [29–32].

In light of this, appropriate OTC products with a reasonable evidence base are a potential avenue for reducing depression, anxiety and insomnia symptoms in later life, and if used effectively, could reduce the need for healthcare professional support. However, despite their commonality, OTC product knowledge is limited in older people [33] and pharmacists [34]. Although some OTC products have been systematically reviewed previously, e.g. melatonin [35], omega-3s [36], vitamin D [37], probiotics [38] or St John’s wort [39], many focus on general adult populations and do not compare across products. It is particularly important to assess products in older people, who may be taking other medications or have comorbid conditions, as surveys suggest concurrent use of OTC medication, herbal medicines, prescription medication and dietary supplements are common [29, 40].

Therefore, we aimed to understand the types and characteristics of oral OTC products (including medication, herbal medicines, homeopathic products and dietary supplements) evaluated in trials for reducing symptoms of depression, anxiety and/or insomnia in older people, to identify potential gaps and challenges in product use.

## Methods

We carried out a scoping review following the Joanna Briggs Institute (JBI) Manual for Evidence Synthesis [41]. A review protocol was developed and uploaded to the Open Science Framework (https://osf.io/rkm57/) before study commencement. The review was performed as part of a larger review focussing on all ages for depression, anxiety, and insomnia. The present review summarises the studies for older adults (60+); for those aged 18–60 separate reviews are being carried out.

### Search strategy and selection criteria

After pilot searches in Medline and Embase to identify suitable keywords and subject headings, we carried out a comprehensive search of CENTRAL, MEDLINE, EMBASE, PsycInfo, CENTRAL and AMED (inception to 19th December 2022), combining terms for (1) OTC products, (2) depression, anxiety, and insomnia, and (3) clinical trial terms, using a validated filter where possible (see Appendix A). General OTC product terms were used as there are no established widely applicable product lists. We also searched the reference list of 10% of reviews (*n* = 200), intending to search more if a large number of relevant new studies were identified, but only two further studies were located. We citation tracked all included studies and followed up all identified protocols and conference abstracts.

Studies were selected based on eligibility criteria in Table 1. In line with scoping review methods, we also outline the context and key definitions in Table 2.

**Table 1:** Inclusion and exclusion criteria

PICOS	Inclusion	Exclusion
**Population**	Adults aged 60+; community-dwelling (including residential care); with symptoms or a diagnosis of insomnia, anxiety, depression, or psychological distress using a questionnaire or diagnostic criteria.	Inpatients; participants with no baseline symptoms or minimum threshold of depression, anxiety, or insomnia (e.g. sleep quality in healthy people), including if it was a symptom of another condition (e.g. substance abuse); other mental health conditions (e.g. stress, dementia).
**Intervention**	Orally administered preparations of OTC medicines, herbal medicines (single or in combination), homeopathic medicines, and dietary supplements (single or in combination), with no restrictions on dosage.	Prescription-only medications (e.g. selective serotonin reuptake inhibitors (SSRIs), Z-drugs); non-oral routes (e.g. topical); other non-pharmacological remedies, devices or mobile applications (e.g. aromatherapy); individualized approaches including consultation with a practitioner; treatment duration < 1 week; products given as part of a larger lifestyle intervention study where effects cannot be separated; tryptophan depletion studies; diet-based interventions.
**Comparator**	Any, including no treatment, placebo or active treatment	No comparator arm
**Outcome**	Depressive, anxiety, or insomnia symptoms using validated questionnaires; remission (resolution of symptoms below a threshold); other mental health symptoms; quality of life; functioning; adherence; adverse events; study withdrawal.	
**Study Design**	Randomised controlled trials (including crossover, cluster, and parallel-group), economic evaluations.	Subgroup analyses (e.g. older people from a larger study of all adults), pre-post test studies involving only a single intervention group, systematic reviews.

**Table 2:** Context

Defining OTC products can be challenging due to the large array of potential products and variation in regulatory frameworks for these across countries (e.g. Kava is available in Australia but not the UK, melatonin is available in France but not the UK, but these products may be purchasable online). As this review is based in the UK, the UK Medicines and Healthcare Regulatory Agency (MHRA) product definitions [42] were used as a starting point. In brief, these are:
• Medicines: substances presented as able to treat or prevent disease in humans, with a view to restoring, correcting or modifying physiological functions through pharmacological activity. OTC medicines includes general sales and pharmacy-only medicines.
• Herbal medicines: the active medicinal product ingredients are herbal substances or preparations only. Usually provided under a Traditional Herbal Regulation registration.
• Dietary supplements: a concentrated source of a vitamin, mineral or other substance, alone or in combination, which has a physiological or nutritional effect, and aims to supplement the normal diet.
• Homeopathic medicine: diluted substances primarily given in tablet form.
However, to ensure findings were more widely applicable, we included products that may be accessed OTC by older people even if their status is not classed in this way in the UK (e.g. melatonin, herbal products without a Traditional Herbal Regulation registration), after discussion with our clinical and PPI team members. We excluded Traditional Chinese Medicine combination products and other clinically unrecognisedproducts (e.g. eel’s head powder) as these are often poorly defined in terms of their composition, are not widely used in a UK setting and would be problematic to assess. To this end, it should also be noted that different product definitions can overlap, particularly dietary supplements (a term used especially in the USA) and herbal (medicinal) products (used commonly in Europe) or listed medicines (Australia), and classification is not always straightforward.

We included studies of all languages and screened using the English abstract and basic translation tools for full texts (e.g. Google Translate). For eligible non-English papers (*n* = 3), one was extracted by a multilingual team member (MH) and the other two were translated using Google Lens, with a multilingual colleague checking key details where uncertain.

Identified records were deduplicated using EndNote (Version X9.3.3) [43]. Given the large volume of hits (*n* = 15,539), five reviewers independently screened a total of 10% titles and abstracts in pairs (RF and SU, SM and AS, SM and VT) using Rayyan [44], and met to discuss and resolve agreements and iteratively refine inclusion criteria as per scoping review methods. When agreements were finalised, each reviewer single screened approximately 20% of the remaining hits, with maybes reviewed by a second reviewer. The same approach was adopted for full text screening, using MS Excel.

### Extracting and presenting the data

SM extracted data using a predefined data extraction form in MS Excel, developed for this study and piloted on two studies. Extracted data included study details, country and setting, sample size, target condition(s), participant characteristics, product type and characteristics, outcomes measured and results. Following data extraction, we descriptively summarised data by condition and product in tables and figures. As quality assessment is not usually part of scoping review methods [41] and our aim was to scope the literature rather than provide a definitive conclusion on the strength of evidence for each product, we did not perform quality assessment.

### Consultation

Two Patient and Public Involvement (PPI) contributors (CV and TC) were involved in conceptualizing the idea, finalising the protocol, choice of products to be included, outcomes to focus on and dissemination.

## Results

### Study selection

**Fig. 1:**
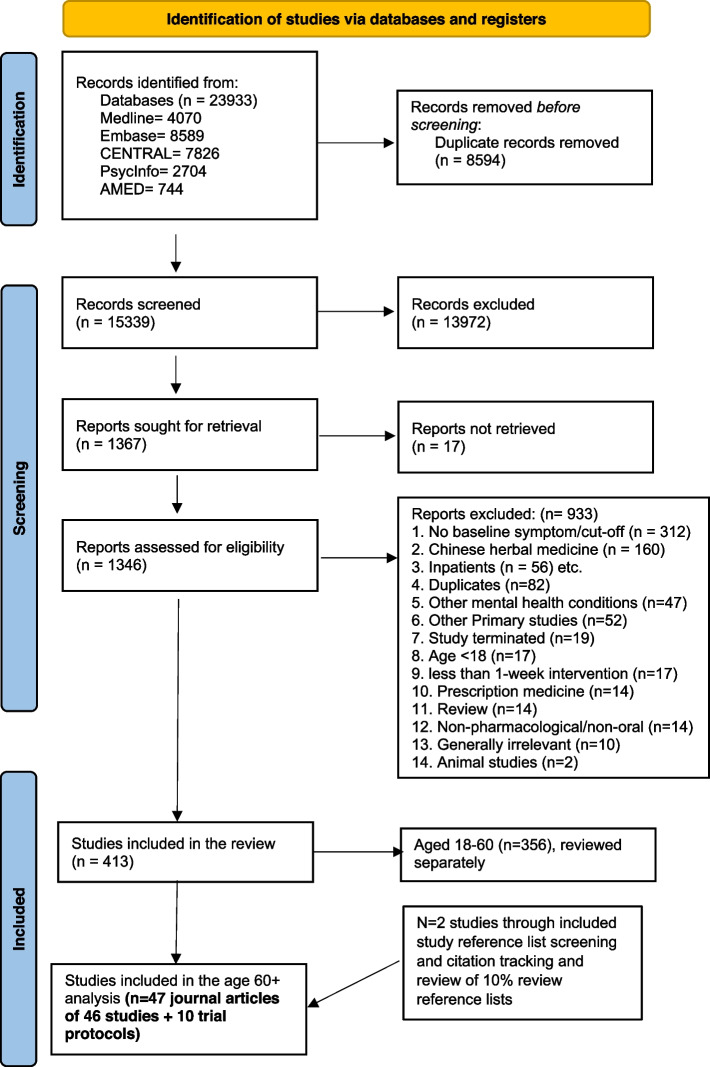
PRISMA Flow Diagram

Out of 23,933 records identified in the overarching review, we screened 15,339 titles and abstracts after deduplication and 1346 full texts, and included 431 papers (see Fig. 1). Reference list screening and citation tracking yielded two additional articles. Fifty-seven papers (47 journal articles of 46 studies and 10 trial protocols) focused on older people (aged 60 + years) were found and are summarised in this paper.

### Characteristics of included studies

#### Study details

Studies were from a range of countries, chiefly Iran (*n* = 8) [45–52], Italy (*n* = 7) [53–59], the United States of America (USA, *n* = 5) [60–64], and China (*n* = 4) [65–68]. Three were multi-country studies (USA and United Kingdom (UK) [69], Germany and Austria [70], and France and Israel [71]). Fifty-seven studies were published in English, two in Chinese [67, 68], and one in German [72]. Thirty-five studies were single centre and 11 multicentre [46, 58, 59, 69, 70, 73–75]. Most were parallel RCTs, with eight crossover trials [60, 63, 64, 70, 74, 76–78], and sample sizes ranged from 12 to 930. Thirty-nine studies were double-blind, with one single-blind study [51], two unclear [46, 67], and five did not report blinding information [52, 57, 68, 79, 80]. There were few noticeable publication trends over time.

#### Participant demographics

Most studies included both men and women, with five focusing exclusively on either male (*n* = 1) [47] or female (*n* = 4) [54, 56, 64, 70] participants. Mean age ranged from 60 to 84 years. Only two studies reported ethnicity information [65, 73]. Twenty-eight studies included participants with no particular comorbidity, and tended to exclude those with other psychiatric disorders, substance abuse, cognitive impairment or significant medical conditions (e.g. cancers, stroke, autoimmune disease). Nineteen studies (41%) included older people with comorbid conditions, including Parkinson’s Disease [48, 81–83], cardiovascular disease [52, 71, 84], Alzheimer’s disease and/or dementia [50, 58, 62, 69], prostate and colorectal cancer [47, 51], a sample with a range of comorbidities [85, 86] and one each of obstructive sleep apnoea [68], haemodialysis patients [75], benzodiazepine withdrawal [87] and post-ischaemic stroke [65].

In most studies, inclusion criteria limited the use of additional depression or insomnia medications (particularly hypnotics), apart from benzodiazepines [61, 76, 87], anti-anxiety or insomnia medication taken for at least one month prior to starting the study [59], venlafaxine [65], variable antidepressants [82] and citalopram [86]. Three trials reported continuing other medication for comorbidities, including chronic heart failure treatment [52], atenolol [71], and acetylcholinesterase inhibitors with or without memantine [69]. Three further studies had trial arms including both an OTC product and a non-pharmacological approach, including a continuous positive airway pressure machine [68], a yoga programme [79] and various lifestyle programmes [77]. Of the studies with concomitant medication, two reported on drug interactions, with no significant interactions [69, 71]. Additional medications taken simultaneously for other conditions were rarely reported unless part of the inclusion criteria.

#### Target condition

**Fig. 2:**
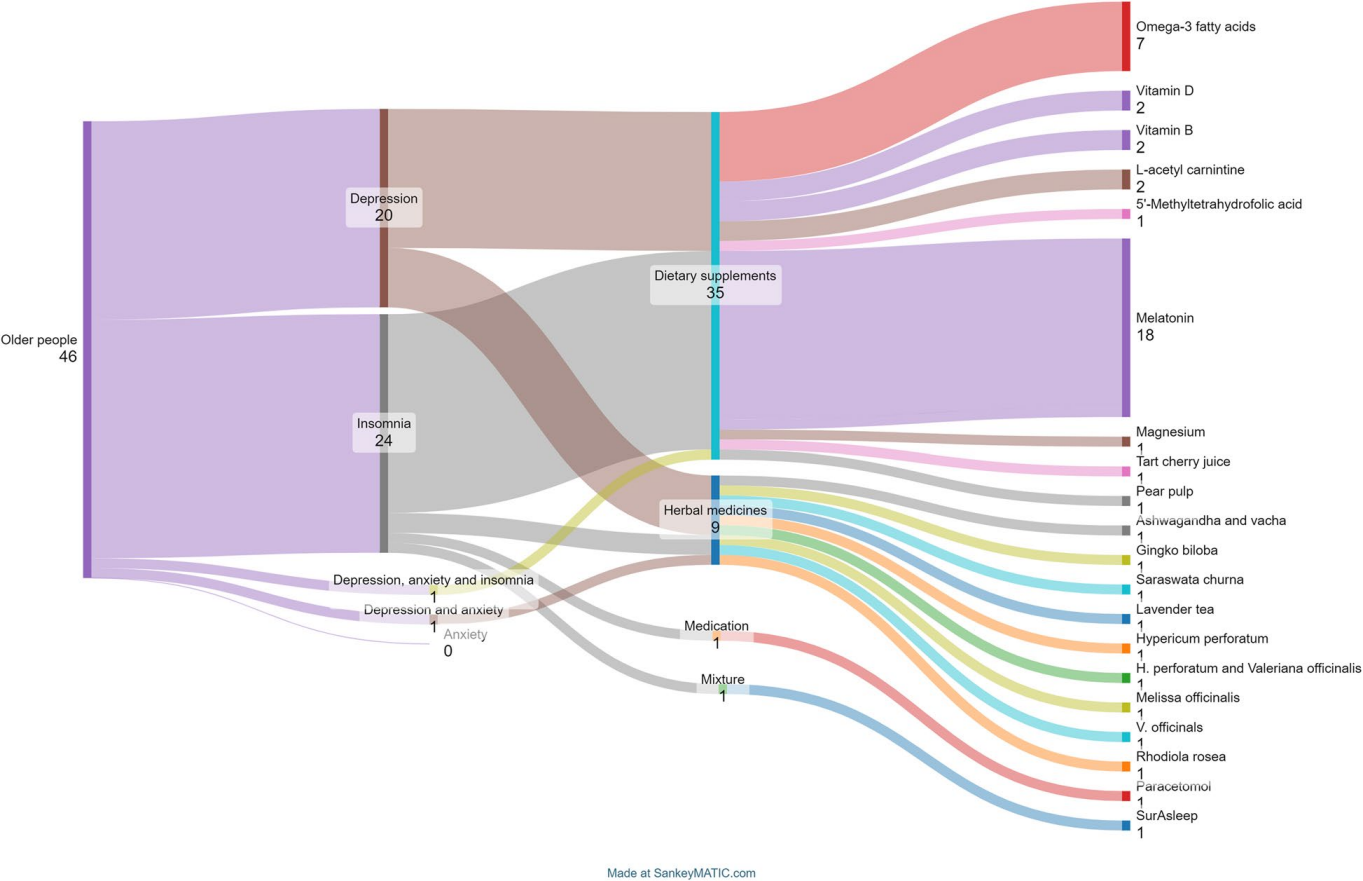
Plot of included studies, conditions and products tested

Included studies focused on insomnia (24/46, 52%), depression (20/46, 43%) and multiple conditions (one all three, one depression plus anxiety) (Fig. 2). No studies focused solely on anxiety.

To assess depressive symptoms at baseline, studies most commonly used the Diagnostic and Statistical Manual of Mental Disorders 4th Edition, Hamilton Depression Rating Scale (HAM-D), and Geriatric Depression Scale (GDS). To assess insomnia, scales such as the Pittsburgh Sleep Quality Index (PSQI), Sleep History Questionnaire, or Insomnia Severity Index (ISI) were used, with a variety of cut-offs. To measure depression as outcome, 10 studies used one outcome measure and 10 studies used multiple measures. The most common measures were the HAM-D (*n* = 10), Beck Depression Inventory (*n* = 5), Clinical Global Impression scale (*n* = 5), GDS (*n* = 4) and Self-rating Depression Scale (*n* = 3), with other measures used in two or fewer studies (see Table 3). To measure insomnia as an outcome, four used a single measure and 20 used multiple measures. These were mainly PSQI (*n* = 10), actigraphy (*n* = 7), sleep diaries (*n* = 7), polysomnography (*n* = 6), Leeds Sleep Evaluation Questionnaire (*n* = 5) and the ISI (*n* = 3). Other measures were used in two or fewer studies (see Table 4).

**Table 3:** Studies assessing OTC products used for depression

Study ID Country, *N* recruited Comorbidities	Product details and duration	Comparator	Depression outcome(s)	Effective on ≥ 1 depression outcome?	Safety
Dietary supplements					
Rondanelli et al., 2011 [53] Italy *N* = 226	Omega-3 long chain polyunsaturated fatty acids (1.67 g EPA and 0.83 g DHA), 2.5 g/day 8 weeks	Placebo (paraffin oil)	GDS	Yes	No side effects.
Rondanelli et al., 2010 [55] Italy *N* = 271	Omega-3 long chain polyunsaturated fatty acids, 2.5 g/day 8 weeks	Placebo (paraffin oil)	GDS	Yes	No serious adverse events
Chang et al., 2020 [84] Taiwan *N* = 237 Comorbidity- Chronic heart disease	Omega-3 long chain polyunsaturated fatty acids (2 g EPA + 1 g DHA)/day 2 weeks	Placebo (soybean oil 3 g/day)	HAM-D BDI	No	Not reported
Pomponi et al., 2014 [83] Italy *N* = 96 Comorbidity-Parkinson’s disease	Omega-3 fatty acid (800 mg/d DHA and 290 mg/d EPA) 6 months	Placebo (vegetable oil)	HAM-D	Yes	Not reported
Rizzo et al. 2012 [54] Italy *N* = 46	Omega-3 oil (1 tbsp or 2.5 g) 1/day 2 months	Placebo (1 tbsp paraffin oil with similar lemony taste)	HAM-D SDS CGI	Yes	No side effects
Tajalizadekhoob et al., 2011 [50] Iran *N* = 66 Comorbidity-dementia	Fish oil (1 g cod liver oil, glycerol, and water; 180 mg EPA and 120 mg DHA) 6 months	Placebo (gelatin capsule containing medium-chain triglycerides, glycerol, and water)	GDS	Yes	Mild effects (gastrointestinal disturbances), settled after a month.
Da Silva et al., 2008 [82] Brazil *N* = 250 Comorbidity- Parkinson’s disease	1. Fish oil capsules (180 mg EPA, 120 mg DHA and tocopherol) 4/day 2. Fish oil plus antidepressant 3 months	1. Placebo x 2 (mineral oil) 2. Placebo plus antidepressant	MADRS CGI BDI	Yes	Not reported
Alavi et al., 2019 [46] Iran *N* = 255	Vitamin D3 50,000 IU weekly at mealtime. 8 weeks	Placebo	GDS	Yes	No side effects were noted.
Koning et al. 2019 [88] Netherlands *N* = 155	Vitamin D3 (400 IU cholecalciferol) 3/day + advice to consume ≥ 3 dairy/day or 500 mg/day calcium tablet for 8 weeks 12 months	Placebo	CES-D MDD incidence (CIDI)	No	No serious adverse effects.
Almeida et al., 2014 [86] Australia *N* = 153 Some comorbidities in sample	Vitamin B (0.5 mg B12, 2 mg folic acid, 25 mg B6) 1 capsule + citalopram 52 weeks	Citalopram + placebo	MDD relapse (DSM-IV) MADRS	Did not enhance, antidepressant treatment, but antidepressant response sustained over 1 year.	No differences in adverse events between study groups
Walker et al., 2012 [77] Australia *N* = 909 2 × 2 × 2 factorial design	1. Vitamin B (400 µg folic acid and 100 µg vitamin B-12) 1/day. After safety review, protocol changed to 2 daily doses (200 µg FA + 50 µg vitamin B-12 each) + physical activity + mental health literacy 2. Supplement + physical activity and pain information 3. Supplement + nutritional information + mental health literacy 4. Supplement + nutritional information + pain information 24 months	4 groups include placebo + other interventions	PHQ-9	Yes	Not reported
Bersani et al., 2013 [59] Italy *N* = 80	L-acetylcarnitine, 1 g, 3/day 7 weeks	Fluoxetine 20 mg/day	HAM-D CGI BDI	Yes	Lesser side effects were noted for L-acetylcarnitine compared to fluoxetine.
Garzya et al., 1990 [57] Italy *N* = 28	L-acetyl-carnitine, 500 mg, 3/day 60 days	Placebo	HAM-D BDI SCAG	Yes	No statistical difference in side effects between groups
Passen et al., 1993 [58] Italy *N* = 120 Comorbidity-dementia	5’-Methyltetrahydrofolic acid 50 mg/day (1 tablet in the morning) 8 weeks	Trazodone 100 mg/day (2 tablets)	HAM-D	Yes	No side effects were noted.
**Herbal products**					
Meleppurakkal et al., 2021 [79] India *N* = 75	1. Ashwagandha (*Withania somnifera* (Linn) Dunal) 3.75 g + Vacha (*Acorus calamus* L.) 250 mg, 4 g powder 2/day with water 2. Yoga + Ashwagandha and Vacha 30 days	Yoga alone	HAM-D	Yes (most significant in group plus yoga)	Not reported
Liang et al., 2019 [65] China *N* = 93 Comorbidity- Ischaemic stroke	Ginkgo (*Gingko biloba* L.*)* 40 mg, 3/day + venlafaxine 75 mg/day 8 weeks	Venlafaxine 75 mg/day only	HAM-D SDS	Yes	Fewer adverse events in experimental group
Tiwari et al., 2011 [80] India *N* = 60	Saraswata Churna (preparation involved Kustha, Ashwagandha, Ajmmoda, Sweta and Krisna Jiraka, Sunthi, Marich, Pipali, Patha, Sankhpuspi, and Vacha powder) 1.5 g, 2/day with 1tsp (5 ml) ghrita & 0.5tsp (2.5 ml) honey, after meals 3 months	Citalopram 20 mg once a day	HAM-D	Yes	No side effects were noted.
Bazrafshan et al., 2020 [49] Iran *N* = 114	Lavender tea (2 g teabag, genus and species not reported) steeped for 10–15 min in 300 mL hot water, 2/day 2 weeks	No treatment	BDI	Yes	No side effects were noted.
Harrer et al., 1999 [70] Germany & Austria *N* = 161	St John’s Wort (*Hypericum perforatum* L. dry extract, LoHyp-57) 200 mg, 2/day 6 weeks	5.6 mg of fluoxetine hydrochloride (equivalent to 5 mg fluoxetine)	HAM-D SDS CGI	St John’s Wort was non-inferior to fluoxetine.	12 adverse drug reactions in LoHyp-57 (6 abandoned treatment) and 17 in fluoxetine (8 abandoned treatment)
Steger et al., 1985 [72] Germany *N* = 100	St John’s Wort (*Hypericum perforatum* L., 90-100 mg standardised to 0.05 mg hypericin*)* and Valerian (*Valeriana officinalis* L. 50 mg 6:1 extract*)* extract Sedariston Konzentrat^®^ 6 weeks	Desipramin hydrochloride 25 mg	CGI D-S	Yes	Occasionally tiredness, dizziness, tachycardia dry mouth - all minor complaints

*BDI* Beck Depression Inventory, CES-D Centre for Epidemiological Studies Depression Scale, *CGI* Clinical global impression, *CIDI* Composite International Diagnostic Interview, *DHA* Docosahexaenoic Acid, *D-S* Depressivitäts-Skala, *DSM-IV* Diagnostic and Statistical Manual of Mental Disorders IV, *EPA* Eicosapentaenoic acid, *GDS* Geriatric Depression Scale, *HAM-D* Hamilton Depression Rating Scale, *MADRS* Montgomery-Asberg Depression Rating Scale, *MDD* major depressive disorder, *IU* international units, *PHQ-9*  9-item Patient Health Questionnaire, *SCAG* Sandoz Clinical Assessment – Geriatric, *SDS* self-rating depression rating scale, *tsp* teaspoon

**Table 4:** Studies evaluating OTC products for insomnia in older people

Study ID Country *N* recruited Comorbidities	Product details	Comparator	Insomnia outcome(s)	Effective for ≥ 1 insomnia outcome?	Safety
Dietary supplements					
Garzon et al., 2019 [76] Spain *N* = 22	Melatonin 5 mg/day at bedtime 8 weeks	Placebo	NHSMI Discontinuation of hypnotic drugs	Yes	Treatment well tolerated; 1 patient had palpitations
Gooneratne et al.,2012 [61] USA *N* = 56	1. 0.1 mg immediate and 0.4 mg controlled release melatonin tablets 30 min before bed 2. 1.0 mg immediate release and 4.0 mg controlled release melatonin 30 min before bed 42 days	Placebo	PSQI PSG	No	No serious adverse events
Wade et al., 2007 [89] Scotland *N* = 523	Prolonged release melatonin 2 mg/day 2 h before bedtime 24 weeks	Placebo	LSEQ (QOS, BFW and total) PSQI (total and Q2 and Q4) QON and QOD (sleep diary) CGI	Yes	1 severe adverse event (emotional distress due to a bereavement) in PR-melatonin group, also nasopharyngitis (*n* = 5) and headache (*n* = 4)
Wade et al., 2010 [73, 85] Scotland *N* = 930 Some comorbidities in sample	Prolonged release melatonin, 2 mg/day, 1–2 h before bed 3 weeks, followed by re-randomisation and 26 weeks extension period	Placebo	Sleep latency (sleep diary) PSQI CGI	Yes	19 drug-related adverse events. No significant difference between the 2 groups, and 1 SUSAR (palpitations in melatonin group)
Wade et al., 2014 [69] UK & USA *N* = 80 Comorbidity- Alzheimer’s disease	Prolonged release melatonin 2 mg/day, 1–2 h before bed. Patients instructed to spend 2 h/day in outdoor daylight. 24 weeks	Placebo	PSQI Sleep diary	Yes	PRM well tolerated, adverse event profile similar to placebo
Lähteenmäki et al.,2013 [87] Finland *N* = 211 Benzodiazepine withdrawal	Controlled release melatonin 2 mg tablet daily 6 months	Placebo	Total BZD withdrawal Reduction in BZD use	No insomnia endpoints (primary outcome benzodiazepine withdrawal)	No serious adverse events in either group
Haimov et al.,1995 [90] Israel *N* = 26 3 arm crossover RCT	1. Sustained-release melatonin 2 mg 2 h before bed 2. Fast-release melatonin 2 mg 2 h before desired bedtime 1 week (2 washout period)	Placebo	Actigraphy	Yes for sustained release vs. placebo, no for fast release vs. placebo	NR
Ahn et al., 2020 [81] South Korea *N* = 34 Comorbidity- Parkinson’s disease	Prolonged-release melatonin 2 mg 1 h before bed 4 weeks	Placebo	PSQI ESS	Yes	No side effects.
Lemoine et al., 2007 [71] France & Israel *N* = 170 Comorbidity- cardiovascular disease	Prolonged-release melatonin. 2 mg 1-2 h before bed and after evening meal 3 weeks	Placebo	LSEQ (QOS, BFW) QON Rebound insomnia Withdrawal following discontinuation	Yes	Low incidence of adverse events and most side-effects were of minor severity
Hadi et al., 2022 [48] Iran *N* = 130 Comorbidity – Parkinson’s disease	Melatonin 3 mg/day 4 weeks	1. Clonazepam 1 mg/day 2. Trazodone 50 mg/day	PSQI	Yes	Mild adverse events reported: clonazepam (*n* = 3), trazodone (*n* = 2), melatonin (*n* = 0)
Luthringer et al.,2009 [91] France *N* = 52	Prolonged release melatonin, 2 mg 2 h before bed and after food 3 weeks	Placebo	PSG LSEQ parameters	Yes	Adverse events reported by 11 patients in each group (most commonly headache), none treatment related.
Hughes et al.,1998 [63] USA *N* = 26 4 group crossover trial	1. 0.5 mg immediate release melatonin taken 30 min before bedtime (+ placebo 4 h after) 2. 0.5 mg continuous release taken 30 min before bedtime (+ placebo 4 h after) 3. 0.5 mg immediate release melatonin 4 h after bedtime (+ placebo 30 min before bed) 2 weeks (2 week washout period)	Placebo capsules (lactose) taken 30 min before and 4 h after bedtime	PSG Actigraphy	Yes	NR
Singer et al., 2003 [62] USA *N* = 244 Comorbidity-Alzheimer’s disease	1. Sustained-release melatonin (2.5 mg) 1 h before bed 2. Immediate release (10 mg) melatonin 1 h before bed 2 months	Placebo	Actigraphy	No	No differences in number, severity, seriousness, or relatedness ratings of spontaneously reported adverse events across the 3 groups
Baskett et al., 2003 [78] New Zealand *N* = 40 Crossover trial	Fast release melatonin, 5 mg at bedtime 4 weeks (4 week washout period)	Placebo	Actigraphy Sleep diary	No	Very few side effects reported, 1 participant excessive drowsiness in both melatonin and placebo groups
Russcher et al., 2013 [75] Netherlands *N* = 67 Comorbidity- Haemodialysis patients	Immediate-release melatonin tablets 3 mg at 10pm 12 months	Placebo	Actigraphy	Yes	NR
Jing-gui et al.,2005 [67] China *N* = 41	Melatonin 2 × 6 mg capsules, 60 min before bed 6 months	Placebo	PSQI ESS PSG	Yes	No serious adverse events occurred
Shahrokhi et al., 2021 [51] Iran *N* = 101 Comorbidity- Colorectal cancer	Melatonin, 2 × 3 mg before bed 30 nights	Zolpidem (2 × 5 mg)	PSQI GSQS	No (similar improvements)	Fatigues (*n* = 2), next morning dizziness (n-1), and GI effects (*n* = 10). No statistical difference between groups.
Abbasi et al., 2012 [45] Iran *N* = 46	Magnesium tablet (414 mg magnesium oxide as 250 mg elemental Magnesium), 500 mg per day 8 weeks	Placebo	ISI Sleep diary	Yes	NR
Pigeon et al., 2010 [60] USA *N* = 15 2 arm crossover trial	Tart cherry juice (TCJ) beverage (CherryPharm, Inc), 1 × 8oz servings 8-10am and 1–2 h before bedtime. 2 weeks (2 week washout period)	Placebo (unsweetened black cheery kool-aid)	ISI Sleep diary	Yes	NR
Rondanelli et al., 2011 [56] Italy *N* = 226	5 mg melatonin, 225 mg magnesium and 11.25 mg zinc conveyed in 100 g pear pulp, once a day 1 h before bedtime. 8 weeks	Placebo (100 g pear pulp alone)	PSQI LSEQ ESS SWAI SDQ Actigraphy	Yes	Patients tolerated the treatment, 2 mild headaches in treatment group
**Herbal supplements**					
Taibi et al., 2009 [64] USA *N* = 18 2 arm crossover trial	Valerian (*Valeriana officinalis* L. root) Nature’s Resource 100 mg softgels standardised to 0.8% valerenic acid, 30 min before bed 2 weeks (2 week washout period)	Placebo (600 mg lactose)	MSQ PSG Sleep diary Actigraphy	No	No serious adverse events occurred
Aliakbari et al., 2018 [52] Iran *N* = 87 Comorbidity- Chronic heart failure	*Melissa officinalis* L. syrup 12 ml/day an hour before going to bed + conventional CHF treatment 1 month	Alprazolam + conventional CHF treatment	PSQI	Yes	NR
**Mixture**					
Xie et al., 2015 [66] China *N* = 100	SurAsleep (calcium, magnesium, valerian root (*Valeriana officinalis* L.), oat straw (*Avena sativa* L.), L-theanine and melatonin) 1 capsule, 30–60 min before bed 12 weeks	Placebo	PSQI Insomnia symptom questionnaire designed for study	Yes	No significant adverse events reported.
**Medication**					
Van de Glind et al., 2014 [74] Netherlands *N* = 61	Acetaminophen 1000 mg/day at bedtime 3 weeks	Placebo	ISI Sleep diary VAS of sleep quality	No	No adverse effects were reported

*BFW* Behaviour following wakening, *BZD*  benzodiazepine, *CGI* Clinical Global Improvement, *ESS* Epworth Sleepiness Scale, *GSQS* Groningen Sleep Quality Scale, *hr* hours, *ISI* Insomnia Severity Index, *min* minutes, *LSEQ* Leeds Sleep Evaluation Questionnaire, *MSQ* Morning Sleep Questionnaire, *NHSMI* Northside Hospital Sleep Medicine Institute Test, *NR* not reported, *PS* polysomnography, *PSQI* Pittsburgh Sleep Quality Index, *QOD* Quality of Day, *QON* Quality of Night, *QOS* Quality of Sleep, *SDQ* Short Insomnia Questionnaire, *SUSAR* Suspected Unexpected Serious Adverse Reaction, *SWAI* Sleep-Wake Activity Inventory, *VAS* visual analogue scale

#### Type of product used

Most studies (*n* = 37/46) evaluated dietary supplements (Fig. 2). Products were given for between two weeks and 12 months and were predominantly used alone (*n* = 39), with nine studies evaluating products alongside another therapy [52, 65, 68, 69, 77, 79, 82, 86, 87]. Thirty-three products were compared to a placebo, eight to an active treatment [48, 51, 52, 59, 65, 70, 72, 80], three to both placebo and active drug [58, 82, 86], one to no treatment [49], and one to yoga [79].

### Depression (*n* = 20 studies)

For depression, 14 trials evaluated five dietary supplements [46, 50, 51, 54, 55, 57–59, 77, 82–84, 86, 88] (Table 3). Omega-3 fatty acid capsules were most frequently evaluated (*n* = 7 studies, sample sizes 46 to 271), either in the form of long-chain polyunsaturated fatty acids (eicosapentaenoic acid (EPA) and docosahexaenoic acid (DHA) [53–55, 83, 84]) or fish oils [50, 82]. Where reported, EPA doses ranged from 180 mg–2 g per day and DHA doses ranged from 120 mg–1 g per day, with supplements taken for between two weeks and six months, indicating a wide dosage range tested. Six out of seven studies comparing omega-3 fatty acids to placebo found a significant improvement in depression symptoms assessed by validated questionnaires, including in people with Parkinson’s disease [82, 83] and dementia [50] but not in people with chronic heart disease [84]. Out of the four studies reporting safety data, two found no side effects and two reported mild gastrointestinal side effects and headache [50, 55].

L-acetyl-carnitine was tested as 3 g/day for seven weeks and 1.5 g/day for 60 days [57, 59]. Despite small studies (*n* = 80 and *n* = 28), both were effective compared to fluoxetine and placebo, respectively, with fewer adverse events than fluoxetine and similar events to placebo [57, 59]. Vitamin D3 showed mixed effects across two studies compared to placebo [46, 88], which may be attributable to large dosage differences (50,000IU weekly vs 1200IU per day), with no serious side effects reported. Vitamin B12 and folic acid effectively reduced depressive symptoms compared to placebo [77], but a combination of B6, B12 and folic acid showed no additional effects alongside citalopram in people with comorbidities [86]. 5’-Methyltetrahydrofolic acid (50 mg/day for 8 weeks) showed no difference to Trazodone in people with dementia, with no side effects noted [58].

Six trials (sample sizes 60–161) evaluated six herbal products [49, 65, 70, 72, 79, 80], including 4 g ashwagandha (*Withania somnifera* (Linn) Dunal) and vacha (*Acorus calamus* L.) [79], Saraswata Churna (a powdered mixture of 13 herbs, 3 g/day) [80], gingko (120 mg/day *Gingko biloba* L.*)* as an adjunct to venlafaxine in people with ischaemic stroke [65], lavender tea (*Lavandula*, species not reported*)* [49], St John’s Wort (*Hypericum perforatum* L. 400 mg/day for six weeks) alone [70], and St John’s Wort + valerian (*Valeriana officinalis* L.) for 6 weeks [72]. All showed positive effects, four compared to prescribed medication, one to yoga [79] and one to no treatment [49]. Lesser and milder adverse events were reported for gingko compared to venlafaxine [65], and slightly fewer adverse reactions were reported for St. John’s Wort than fluoxetine [70]. No side effects were noted for lavender and sarasvata churna [49, 80]. No trials were found for medication or homeopathic medicines.

### Insomnia (*n* = 24)

Melatonin (*n* = 17 studies in 18 publications, samples sizes 22 to 930) was the most commonly evaluated dietary supplement, over time periods of 1 week to 12 months. Sustained-release forms were assessed in 10 studies (mostly 2 mg, but varying from 0.5 to 4 mg doses taken before bedtime), and nine showed effects on one or more sleep outcomes vs. placebo [63, 69, 71, 81, 85, 89, 90], whilst one showed no effects [62] and one only measured safety outcomes [87]. Immediate release melatonin was evaluated in five studies (0.5-5 mg before bed), three of which found no effects [62, 78, 90] and two found effects [63, 75]. One evaluated a mix of immediate and sustained release melatonin at two dosage levels and found no effects vs. placebo [61]. Four did not report the form and used doses of 3-6 mg, and found positive effects vs. placebo [67, 76] and better or similar effects to prescribed drugs [48, 51]. Effects were found in both general older populations and people with cardiovascular disease [71], Parkinson’s disease [81] and on haemodialysis [75], with mixed effects in Alzheimer’s disease [62, 69], and similar effects to Zolpidem in people with colorectal cancer [51]. Thirteen out of 18 studies reported safety data and indicated melatonin was well tolerated in older people with no or only mild AEs and few SAEs [48, 51, 62, 69, 71, 76, 78, 85, 89].

Three other dietary supplements were evaluated. Magnesium (500 mg/day for 8 weeks) reduced insomnia compared to a placebo in 46 people (no safety data) [45]. Tart cherry juice (8oz twice/day for two weeks) reduced insomnia severity, but had no effect on other sleep parameters and did not report safety [60]. Pear pulp with added magnesium, melatonin and zinc reduced all insomnia-related parameters compared to pear pulp alone and was tolerated well [56].

For herbal products, Valerian (*Valeriana officinalis* L. root 100 mg as a soft gel) was ineffective compared to placebo, with no difference in side effects [64]. *Melissa officinalis* L. syrup (12 ml/day) showed similar effects to alprazolam, and had significantly shorter time taken to fall asleep, but did not report safety [52]. SurAsleep (a mixture of herbs and dietary supplements) was taken for 12 weeks and how effects vs. placebo in 100 people, with no significant adverse events reported [66].

For OTC medication, 1000 mg paracetamol at bedtime for three weeks did not improve sleep compared to a placebo in 61 people, and no safety data were reported [74].

### Combination of conditions (*n* = 2, Table 5)

Melatonin (3 mg) was also evaluated for depression, anxiety, and insomnia in 63 people [47]. It significantly improved sleep scores but not depression or anxiety, but excessive daytime sleepiness was a side effect [47]. A second study evaluated rhodiola (*Rhodiola rosea* L.) for depression and anxiety symptoms in 90 people with comorbid obstructive sleep apnoea [68]. When used with a continuous positive airway pressure (CPAP) machine, rhodiola reduced depression and anxiety, but not alone. No safety information was provided.

**Table 5:** Studies assessing OTC products for combinations of conditions

Study Details	Product used	Comparator used	Measure(s) used	Effective?	Side effects
Etedali et al., 2022 [47] Iran *N* = 63 Comorbidity-prostate cancer	Melatonin 3 mg twice a day 4 weeks	No treatment	PSQI HAM-A BDI	Yes (for insomnia, not depression or anxiety)	Excessive daytime sleepiness with melatonin
Yu et al., 2019 [68] China *N* = 90 Comorbidity- Obstructive sleep apnoea	1. *Rhodiola rosea* L. 0.6 g twice a day 2. *Rhodiola rosea* L. + CPAP machine 3 months	CPAP machine only	SDS SAS	Yes for depression and anxiety when used with CPAP but not alone	Not reported

*BDI* Beck Depression Inventory, *HAM-A* Hamilton Anxiety Rating Scale, *PSQI* Pittsburgh Sleep Quality Index, *SAS* Self-rating Anxiety Scale, *SDS* Self-rating Depression Scale

### Economic evaluations

No economic evaluations of OTC products were found.

### Protocols

We identified 10 protocols without a related publication – eight were listed as completed and two are ongoing. The two ongoing studies are targeted at insomnia and depression in older people and evaluate a dietary supplement containing green tea, chicory and collagen, and Tiryaqe wabai (Unani herbal preparation). Three completed trials were targeted at depression, evaluating vitamin C, omega-3 fatty acid, and a combination of folic acid plus omega-3 fatty acids. Four protocols evaluated the effects of melatonin (*n* = 3) and saffron (*n* = 1) for insomnia (Appendix B). One trial evaluated the effects of prebiotic and probiotic supplements for anxiety.

## Discussion

In this scoping review, we found that surprisingly few OTC products were investigated for depression, anxiety or insomnia in older people – only 46 studies of 21 products were found – and these were mostly tested in those without comorbidities, or with a single specific comorbidity. Given 65% of people aged 65–84 have multiple long term conditions, rising to 82% in the over 85s [92], most trials have been conducted in unrepresentative populations. Few products had multiple trials, apart from omega-3 fatty acids (*n* = 7), which had more substantive evidence for depressive symptoms, and melatonin (*n* = 17) which had more substantive evidence for insomnia. Most products had a good safety profile where this was reported, but 13 studies did not report safety data. There was a clear gap in the evidence base in products for anxiety in older people, OTC medications such as antihistamines and homeopathic products.

The products with the most trials do represent some of the most popular products. Omega 3s are commonly used by older people [27, 33]. Systematic reviews of omega-3s for depression in older people find that effects are likely to be dose-dependent and consistent across physical health conditions, and were from good quality studies [36, 93]. A recent meta-analysis of melatonin showed improved sleep quality in a meta-analysis of 23 trials, of mostly good quality, with no differences for people aged over 65 [94]. Although not available OTC in the UK, US melatonin sales to all ages almost tripled between 2016 and 2020, suggesting it is popular [95].

However, there were clear discrepancies between other OTC products tested and used by older people. OTC antihistamines are commonly used to induce sleep in the UK [96], but no studies were found in older adults. One crossover trial located separately found diphenhydramine for two weeks reduced number of awakenings in 25 older people, compared to placebo, but no other sleep parameters, and was less effective than temazepam [97]. Positive effects were found for 50 mg for 1–2 weeks in younger people but greater adverse effects than placebo and a valerian-hops combination [98, 99]. Our review found one paracetamol trial – whilst not indicated for sleep, the authors’ rationale for evaluating this was observing patients self-medicating using this for sleep in geriatric clinical practice [74].

Other commonly used herbal supplements requiring further research in older people include Valerian, Nytol herbal (hops, valerian and passionflower) and St John’s Wort [29], which have little evidence in older people. Products for anxiety in later life are a particularly large gap in the literature requiring further research. Later-life anxiety is less well understood than depression, but is common and is associated with adverse outcomes such as cognitive impairment [9]. Promising anxiolytic herbal products identified in a network meta-analysis of herbal products in younger age groups include Silexan (a lavender oil preparation), kava, passionflower, saffron, gingko and ashwagandha [100]. These should be evaluated in older samples. At present, further comparative reviews of OTC products for older people are unlikely to yield much useful information given the paucity of studies, and further primary studies are needed on promising products.

People aged 65 to 85 are typically living with on average 2.6 long-term conditions [92], which are likely to require medication. Polypharmacy is extremely common and increases the risk of drug-related harm [101]. Future trials need to take a more pragmatic approach and include older people with comorbidities and/or taking prescription medication [102]. Only nine studies evaluated products as adjunct therapies. Real-life supplement use is also likely to involve taking multiple OTC products simultaneously [27]. Whilst some trials evaluated products containing multiple ingredients, further evaluation of combinations are needed. Few surveys assess older people’s usage of OTC products for specific problems; preparatory work should determine what is commonly used prior to new trials.

Financial aspects of OTC product usage are also rarely considered, despite costs for individuals. Our review found no studies on this. It is unclear if OTC product usage reduces healthcare usage through better self-management, reduced medication costs, and reduced risk of adverse events; increases healthcare usage through potential risk or interactions; or has no impact. As UK policy is directing more people towards self-management and seeking pharmacy support [103], this remains an avenue for further exploration.

Strengths of this review include following established guidelines [41], a thorough and iterative search strategy and inclusion of all languages. Due to the large volume of hits, we were only able to dual screen 10% titles, abstracts and full texts. It was particularly challenging to define OTC products in a global context, and it is possible some promising products were missed. Herbal products and dietary supplements are also not necessarily consistent across supplier and product, or may straddle the definition of food and supplements (e.g. tart cherry juice). We employed an inclusive approach in this review to encompass a wide range of products, as advised by our public contributors, but different implications may arise from this review in different contexts.

## Conclusion

UK NICE guidelines do not currently recommend OTC products for insomnia or depression [104, 105]. For older people, based on the current review this recommendation seems reasonable. However, there are obviously high OTC product usage levels and some which have a more substantial volume of evidence. Our review suggests that future primary studies should focus on widely used products, with a more pragmatic approach to testing, including populations similar to those who would use them in practice. There is a particularly large gap in evaluating products for anxiety in older people; future research should prioritise this.

### Supplementary Information

Supplementary Material 1.

Supplementary Material 2.

## Data Availability

The data extraction form upon which the review results are based can be obtained from the authors upon reasonable request.
