# Supplementary material for: A scoping review of over-the-counter products for depression, anxiety and insomnia in older people
Source: BMC Complement Med Ther. 2024 Jul 20;24:275. doi: 10.1186/s12906-024-04585-0 (PMC11264918; doi:10.1186/s12906-024-04585-0)
Supplement: Supplementary file 1 — Supplementary Material 1. [file 12906_2024_4585_MOESM1_ESM.docx]

**Supplementary File 1: Search Log**

| **Database** | **String** | **Total Hits** |
| --- | --- | --- |
| **Medline** | 1 (anxi* or (anxiety adj1 disorder) or (stress* adj1 (psychological or emotional)) or panic attack or depress* or mental health or dysthymia or (panic adj1 disorder) or (sleep adj1 disorder) or insomnia or (mood adj1 disorder) or (psychological adj1 distress)).ti,ab,kw.  2 mental health/ or anxiety disorders/ or mood disorders/ or Anxiety/ or exp Depression/ or exp "Sleep Initiation and Maintenance Disorders"/  3 1 or 2  4 ("Over-the-counter" or OTC or (over the counter adj1 (medic* or drug or product)) or "General sales" or "pharmacy only" or Non-prescription or Self-medication or Self-prescription or anti-histamines or (botanical or herb* or "medicinal plant" or fungus or medicinal mushroom or herbal tea or plant extract or flower extract or root extract or seed extract or "bach flower" or "natural product" or CBD or cannabidiol) or ("nutritional supplement" or "dietary supplement" or vitamins or minerals or "amino acids" or "essential fatty acids" or "omega-3 fatty acid" or 5-Htp or 5-Hydroxytryptophan or tryptophan or probiotic or prebiotic or melatonin) or ((Traditional adj1 medicine) or (Chinese adj1 medicine) or ayurv* or homeopathy or "homeopathic medicine" or phytotherap* or nutraceutical or "herbal medicine")).ti,ab,kw.  5 exp Nonprescription Drugs/ or plant extracts/ or teas, herbal/ or Plants, Medicinal/ or Cannabidiol/ or Dietary Supplements/ or exp Vitamins/ or Vitamin D/ or Vitamin E/ or Folic Acid/ or magnesium compounds/ or minerals/ or zinc compounds/ or amino acids/ or Tryptophan/ or Fatty Acids/ or homeopathy/ or exp medicine, traditional/ or Melatonin/  6 4 or 5  7 exp animals/ not humans.sh.  8 (randomized controlled trial or controlled clinical trial).pt. or randomized.ab. or placebo.ab. or clinical trials as topic.sh. or randomly.ab. or trial.ti.  9 8 not 7  10 (cost-effectiveness or (cost adj1 effectiveness adj1 analysis) or (cost adj1 benefit adj1 analysis) or "economic evaluations").ti,ab,kw.  11 Cost-Benefit Analysis/  12 9 or 10 or 11  13 3 and 6 and 12  14 limit 13 to humans | **4070** |
| **Embase**  **(Embase classic + Embase)** | 1 (anxi* or (anxiety adj1 disorder) or (stress* adj1 (psychological or emotional)) or panic attack or depress* or dysthymia or (panic adj1 disorder) or (sleep adj1 disorder) or insomnia or (mood adj1 disorder) or (psychological adj1 distress)).ti,ab,kw.  2 anxiety disorder/ or exp anxiety/ or exp depression/ or exp insomnia/  3 1 or 2  4 ("Over-the-counter" or OTC or (over the counter adj1 (medic* or drug or product)) or "General sales" or "pharmacy only" or Non-prescription or Self-medication or Self-prescription or (botanical or herb* or "medicinal plant" or fungus or medicinal mushroom or herbal tea or plant extract or flower extract or root extract or seed extract or "bach flower" or "natural product" or CBD or cannabidiol) or ("nutritional supplement" or "dietary supplement" or vitamins or minerals or "amino acids" or "essential fatty acids" or "omega-3 fatty acid" or 5-Htp or 5-Hydroxytryptophan or tryptophan or probiotic or prebiotic or melatonin) or ((Traditional adj1 medicine) or (Chinese adj1 medicine) or ayurv* or homeopathy or "homeopathic medicine" or phytotherap* or nutraceutical or "herbal medicine")).ti,ab,kw.  5 exp Nonprescription Drugs/ or exp plant extract/ or exp medicinal plant/ or herbal tea/ or exp herbal medicine/ or cannabidiol/ or exp diet supplementation/ or exp vitamin/ or exp mineral/ or zinc/ or 5 hydroxytryptophan/ or omega 3 fatty acid/ or exp fatty acid/ or exp traditional medicine/ or homeopathy/ or melatonin/  6 4 or 5  7 (randomized controlled trial or controlled clinical trial).pt. or randomized.ab. or placebo.ab. or clinical trials as topic.sh. or randomly.ab. or trial.ti.  8 (((((((((((random* adj1 sampl* adj8 (cross section* or questionnaire* or survey or surveys or database or databases)).ti,ab,tt. not (comparative study/ or controlled study/ or randomised controlled.ti,ab,tt. or randomized controlled.ti,ab,tt. or randomly assigned.ti,ab,tt.)) or Cross-sectional study/) not (randomized controlled trial/ or controlled clinical study/ or controlled study/ or randomi?ed controlled.ti,ab. or control group$1.ti,ab.)) or (((case adj control$) and random$) not randomi?ed controlled).ti,ab. or (Systematic review not (trial or study)).ti. or (nonrandom$ not random$).ti,ab. or "Random field$".ti,ab. or (random cluster adj3 sampl$).ti,ab. or (review.ab. and review.pt.)) not trial.ti.) or "we searched".ab.) and (review.ti,tt. or review.pt.)) or "update review".ab. or (databases adj4 searched).ab. or (rat or rats or mouse or mice or swine or porcine or murine or sheep or lambs or pigs or piglets or rabbit or rabbits or cat or cats or dog or dogs or cattle or bovine or monkey or monkeys or trout or marmoset$1).ti.) and animal experiment/) or Animal experiment/) not (human experiment/ or human/)  9 7 not 8  10 (cost-effectiveness or (cost adj1 effectiveness adj1 analysis) or (cost adj1 benefit adj1 analysis) or "economic evaluations").ti,ab,kw. or exp economic evaluation/  11 9 or 10  12 3 and 6 and 11  13 limit 12 to human | **8589** |
| **PsychINFO** | 1 (anxi* or (anxiety adj1 disorder) or (stress* adj1 (psychological or emotional)) or panic attack or depress* or mental health or dysthymia or (panic adj1 disorder) or (sleep adj1 disorder) or insomnia or (mood adj1 disorder) or (psychological adj1 distress)).ti,ab,hw.  2 exp "depression (emotion)"/ or atypical depression/ or exp late life depression/ or "long-term depression (neuronal)"/ or exp postpartum depression/ or exp anxiety/ or exp insomnia/  3 ("Over-the-counter" or OTC or (over the counter adj1 (medic* or drug or product)) or "General sales" or "pharmacy only" or Non-prescription or Self-medication or Self-prescription or anti-histamines or (botanical or herb* or "medicinal plant" or fungus or medicinal mushroom or herbal tea or plant extract or flower extract or root extract or seed extract or "bach flower" or "natural product" or CBD or cannabidiol) or ("nutritional supplement" or "dietary supplement" or vitamins or minerals or "amino acids" or "essential fatty acids" or "omega-3 fatty acid" or 5-Htp or 5-Hydroxytryptophan or tryptophan or probiotic or prebiotic or melatonin) or ((Traditional adj1 medicine) or (Chinese adj1 medicine) or ayurv* or homeopathy or "homeopathic medicine" or phytotherap* or nutraceutical or "herbal medicine")).ti,ab,hw.  4 exp nonprescription drugs/ or self-medication/ or histamine/ or antihistaminic drugs/ or exp "medicinal herbs and plants"/ or exp dietary supplements/ or exp vitamins/ or exp alternative medicine/ or amino acids/ or exp cannabidiol/ or exp fatty acids/ or exp tryptophan/ or exp Melatonin/  5 1 or 2  6 3 or 4  7 (cost-effectiveness or (cost adj1 effectiveness adj1 analysis) or (cost adj1 benefit adj1 analysis) or "economic evaluations").ti,ab,hw.  8 exp "costs and cost analysis"/  9 7 or 8  10 Treatment Effectiveness Evaluation/ or exp Treatment Outcomes/ or Placebo/ or Followup Studies/ or placebo*.ti,ab,hw. or random*.ti,ab,hw. or "comparative stud*".ti,ab,hw. or (clinical adj3 trial*).ti,ab,hw. or (research adj3 design).ti,ab,hw. or (evaluat* adj3 stud*).ti,ab,hw. or ((singl* or doubl* or trebl* or tripl*) adj3 (blind* or mask*)).ti,ab,hw.  11 exp animals/ not humans.sh.  12 10 not 11  13 9 or 12  14 5 and 6 and 13  15 limit 14 to human | **2704** |
| **AMED** | 1 exp Psychology/ or exp Anxiety disorders/ or Heart rate/ or exp Affective disorders/ or emotions/ or affect/ or anxiety/ or fear/ or panic/ or loneliness.mp. or exp Depression/ or Agoraphobia/ or sleep disorders/ or insomnia/  2 (anxi* or (anxiety adj1 disorder) or (panic adj1 attack) or depress* or (mental adj1 health) or dysthymia or (panic adj1 disorder) or (sleep adj1 disorder) or insomnia or (mood adj1 disorder) or (psychological adj1 distress) or (stress* adj1 (psychological or emotional))).ti,ab,hw.  3 1 or 2  4 complementary therapies/ or herbalism/ or exp homeopathy/ or exp phytotherapy/ or exp traditional medicine/ or exp Herbal drugs/ or self care/ or exp plants medicinal/ or exp plant extracts/ or exp herbs/ or exp Fungi/ or tea/ or Flowers/ or exp roots/ or fruit/ or seeds/ or exp homeopathic drugs/ or bach flower remedies/ or cannabinoids/ or cannabis/ or dietary supplements/ or exp vitamins/ or exp minerals/ or exp amino acids/ or lipids/ or fatty acids/ or Fish oils/ or exp traditional medicine/ or ethnopharmacology/ or tryptophan/ or histamine/ or serotonin/ or exp food additives/ or probiotics/ 78705  5 ("Over-the-counter" or OTC or (over the counter adj1 (medic* or drug or product)) or "General sales" or "pharmacy only" or Non-prescription or Self-medication or Self-prescription or anti-histamines or botanical herb* or medicinal plant or fungus or medicinal mushroom or herbal tea or plant extract or flower extract or root extract or seed extract or bach flower or natural product or CBD or cannabidiol or nutritional supplement or dietary supplement or vitamins minerals or amino acids or essential fatty acids or omega-3 fatty acid or 5-Htp or 5-Hydroxytryptophan or tryptophan or probiotic or prebiotic or melatonin or Traditional medicine or Chinese medicine or ayurv* or homeopathy or homeopathic medicine or phytotherap* or nutraceutical or herbal medicine).ti,ab,hw.  6 4 or 5  7 exp animals/ not humans.sh.  8 Trials.mp. or randomized controlled trial.pt. or exp clinical trials/ or Double blind method/ or randomized controlled trial.pt. or controlled clinical trial.pt. or randomized.ab. or placebo.ab. or clinical trials.sh. or trial.ti. or randomly.ab.  9 8 not 7  10 Economics/ or "Cost benefit analysis"/ or (cost-effectiveness or (cost adj1 effectiveness adj1 analysis) or (cost adj1 benefit adj1 analysis) or "economic evaluations").ti,ab,hw.  11 9 or 10  12 3 and 6 and 11 | **744** |
| **CENTRAL** | #1 ((anxi* or ("anxiety disorder") or (stress* near/1 (psychological or emotional)) or "panic attack" or depress* or "mental health" or dysthymia or "panic disorder" or "sleep disorder" or insomnia or "mood disorder" or "psychological distress")):ti,ab,kw (Word variations have been searched)  #2 MeSH descriptor: [Anxiety Disorders] this term only  #3 MeSH descriptor: [Depressive Disorder] explode all trees  #4 MeSH descriptor: [Sleep Initiation and Maintenance Disorders] explode all trees  #5 MeSH descriptor: [Stress, Psychological] this term only  #6 #1 or #2 or #3 or #4 or #5  #7 (("Over-the-counter" or OTC or (over the counter near/1 (medic* or drug or product)) or "General sales" or "pharmacy only" or "Non-prescription" or "Self-medication" or "Self-prescription" or "anti-histamines")):ti,ab,kw (Word variations have been searched)  #8 MeSH descriptor: [Nonprescription Drugs] explode all trees  #9 ((botanical or herb* or "medicinal plant" or fungus or "medicinal mushroom" or "herbal tea" or "plant extract" or "flower extract" or "root extract" or "seed extract" or "bach flower" or "natural product" or CBD or cannabidiol)):ti,ab,kw (Word variations have been searched)  #10 MeSH descriptor: [Herbal Medicine] this term only  #11 MeSH descriptor: [Plant Extracts] explode all trees  #12 MeSH descriptor: [Plants, Medicinal] this term only  #13 MeSH descriptor: [Fungi] this term only  #14 MeSH descriptor: [Teas, Herbal] this term only  #15 MeSH descriptor: [Cannabidiol] this term only  #16 (("nutritional supplement" or "dietary supplement" or vitamins or minerals or "amino acids" or "essential fatty acids" or "omega-3 fatty acid" or "5-Htp" or "5-Hydroxytryptophan" or tryptophan or probiotic or prebiotic or melatonin)):ti,ab,kw (Word variations have been searched)  #17 MeSH descriptor: [Dietary Supplements] this term only  #18 MeSH descriptor: [Vitamins] this term only  #19 MeSH descriptor: [Minerals] explode all trees  #20 MeSH descriptor: [Magnesium] this term only  #21 MeSH descriptor: [Zinc Compounds] this term only  #22 MeSH descriptor: [Amino Acids] this term only  #23 MeSH descriptor: [5-Hydroxytryptophan] explode all trees  #24 MeSH descriptor: [Fatty Acids, Essential] this term only  #25 MeSH descriptor: [Medicine, Traditional] explode all trees  #26 MeSH descriptor: [Phytotherapy] this term only  #27 MeSH descriptor: [Homeopathy] this term only  #28 MeSH descriptor: [Melatonin] this term only  #29 #7 or #8 or #9 or #10 or #11 or #12 or #13 or #14 or #15 or #16 or #17 or #18 or #19 or #20 or #21 or #22 or #23 or #24 or #25 or #26 or #27 or #28  #30 #6 and #28 | **7826** |
