# Supplementary material for: A scoping review of over-the-counter products for depression, anxiety and insomnia in older people
Source: BMC Complement Med Ther. 2024 Jul 20;24:275. doi: 10.1186/s12906-024-04585-0 (PMC11264918; doi:10.1186/s12906-024-04585-0)
Supplement: Supplementary file 2 — Supplementary Material 2. [file 12906_2024_4585_MOESM2_ESM.docx]

**Supplementary File 2: Protocols included**

| **Protocol title** | **Target Condition** | **Product used** | **Web link** |
| --- | --- | --- | --- |
| Effectiveness of Ascorbic Acid and Tocopherol for Depression in Elderly | Depression | Ascorbic Acid and Tocopherol | https://clinicaltrials.gov/ct2/show/NCT02793648 |
| Folic Acid and Omega -3 Fatty Acid Supplementation in Depressed Older Adults | Depression | Folic Acid and Omega -3 Fatty Acid | https://clinicaltrials.gov/ct2/show/NCT00480207 |
| The Beyond Ageing Project: phase II: a selective prevention trial using novel pharmacotherapies in an older age cohort at risk for depression | Depression | Omega-3 fatty acids | https://pubmed.ncbi.nlm.nih.gov/26037484/ |
| The effect of herbal medicine (Tiryaqe wabai) on anxiety, depression and sleep in the elderly | Depression | Tiryaqe wabai | https://trialsearch.who.int/Trial2.aspx?TrialID=IRCT20211017052785N2 |
| Melatonin 3mg and 5mg Compared to Cognitive Behavioral Therapy for Insomnia (CBT-I) in the Treatment of Insomnia | Insomnia | Melatonin | https://clinicaltrials.gov/ct2/show/NCT02798367 |
| Exploration of Relationships Between Sleep, Gut Health and Cognition | Insomnia | Saffron | https://clinicaltrials.gov/ct2/show/NCT05315986 |
| 1.5 versus 3.0 mg APL510 to normalise sleep patterns in elderly subjects with insomnia | Insomnia | Melatonin | https://trialsearch.who.int/Trial2.aspx?TrialID=ISRCTN82088636 |
| A double-blind, parallel group, randomised, placebo controlled study of the efficacy and safety of circadin® 2mg in the improvement of sleep quality in patients with insomnia aged 55-80 years | Insomnia | Melatonin | https://trialsearch.who.int/Trial2.aspx?TrialID=EUCTR2004%E2%80%90000914%E2%80%9037%E2%80%90GB |
| Effect of a Dietary Supplement on Quality of Life | Insomnia | Green tea extract, Chicory extract, and collagen peptides | https://clinicaltrials.gov/ct2/show/NCT05323084 |
| Anti-anxiety Biotics for Breast Cancer Survivors | Anxiety | Probiotic plus prebiotic supplement | https://clinicaltrials.gov/ct2/show/NCT04784182 |
